# The Relationship between Binge Eating Disorder and Suicidality: A Systematic Review

**DOI:** 10.3389/fpsyg.2017.02125

**Published:** 2017-12-05

**Authors:** Chiara Conti, Roberta Lanzara, Mattia Scipioni, Marzia Iasenza, Maria T. Guagnano, Mario Fulcheri

**Affiliations:** ^1^Department of Psychological, Health, and Territorial Sciences, University “G. d'Annunzio” Chieti-Pescara, Chieti, Italy; ^2^Department of Medicine and Aging, University “G. d'Annunzio” Chieti-Pescara, Chieti, Italy

**Keywords:** binge eating disorder, suicidal behavior, suicidal ideation, suicide attempt, suicidality

## Abstract

**Background:** We carried out a systematic review analyzing the relation between binge eating disorder (BED), a recent addition to the eating disorders in DSM-5, and suicidality (i.e., suicidal ideation or attempted and/or committed suicide) by synthesizing the relevant studies' qualitative data.

**Methods:** We conducted, according to PRISMA guidelines, a systematic search of the literature on PubMed, Scopus, ISI Web of Science, PsycINFO, Google Scholar, and ScienceDirect. Search terms were “binge eating disorder” combined with the “AND” Boolean operator and “suicid^*^.”

**Results:** The initial search identified 4,014 records, of which 17 research reports met the predefined inclusion criteria and were analyzed. BED was found to be significantly associated with a marked increase in suicidal behaviors and suicidal ideation (SI). The presence and severity of BED were found to be relevant predictive factors for suicidality, notably in association with mood disorders and specific psychological features, while a high body mass index (BMI) did not always affect suicidality. BED has usually been associated with suicide risk, particularly when occurring with another psychiatric disorder and/or in an adolescent population.

**Conclusion:** Pursuant to these findings, it is necessary to consider both dysfunctional eating behavior and related psychopathological factors that may induce SI and suicidal behavior in BED, aiming to identify patients and subgroups of patients needing greater clinical psychological attention to most effectively prevent and treat suicidality.

## Introduction

Binge eating disorder (BED) is a severe and life-threatening eating disorder (ED) associated with adverse physical consequences, high rates of psychopathology, and emotional and mood dysregulation (Grucza et al., 2007; Musci et al., 2014).

In the most recently released edition of the Diagnostic and Statistical Manual of Mental Disorders, 5th Edition (DSM-5), BED is characterized by recurrent episodes of binge eating, defined by an objective overconsumption of food and a sense of loss of control, without the compensatory behaviors that define bulimia nervosa. In DSM IV-Text Revision (DSM-IV-TR), BED was not considered an established diagnosis and was, instead, included as a provisional diagnosis in Appendix B (American Psychiatric Association, 2013).

It is widely recognized that individuals with EDs show high rates of suicidality, which comprises completed suicide, suicide attempt (SA), and suicidal ideation (SI) (Harris and Barraclough, 1998; Pompili et al., 2006; Carano et al., 2012; Dooley-Hash et al., 2012). SA and SI are two of the best predictors of suicide completion (Jenkins et al., 2002). SA is defined as one suicide attempt with definite suicidal or life-threatening intention, whereas, SI is marked by recurrent suicidal thoughts and suicide methods planning (Laakso et al., 2013). Suicidality is also common in individuals with BED, with lifetime SI estimates ranging from 26.3 to 51.7% (Ackard et al., 2003; Portzky et al., 2014), and lifetime SA estimates ranging from 2.3 to 34% (Suokas et al., 2014; McElroy et al., 2016). However, few studies (Preti et al., 2011) have reported exhaustive data on suicide risk among patients with BED.

Suicide is one of the highest public health priorities worldwide. The World Health Organization's objectives for suicide prevention emphasize identification of high-risk groups (World Health Organization, 2012). It is important to recognize the clinical features associated with suicide risk in patients with BED, both to realize interventions to prevent suicidal behaviors and to treat clinical risk factors underlying suicide. Therefore, we performed a systematic review with the aim of providing new insights on the clinical characteristics of the BED-suicide risk association.

## Materials and methods

### Eligibility criteria

Eligible articles included all English-language papers published in peer-reviewed journals, reporting data on the presence of SI or attempted and/or committed suicide in a sample of adult or adolescent individuals diagnosed with BED, according to the research criteria of DSM-IV-TR or DSM-5. When a title or abstract seemed to describe a study eligible for inclusion, the full text was examined to consider its relevance according to the inclusion criteria. Reviews, meta-analyses, commentaries, letters to the editor, books or book chapters, abstracts, and clearly irrelevant papers were excluded.

### Information sources and searches

This systematic review was conducted according to the Preferred Reporting Items for Systematic Reviews and Meta-Analyses (PRISMA) guidelines (Liberati et al., 2009). PubMed, Scopus, ScienceDirect, ISI Web of Science, PsycINFO, and Google Scholar databases were systematically searched in February 2017 using the following keywords: “binge eating disorder” AND (i.e., Boolean operator) “suicid^*^” [All Fields]. After performing the initial search, duplicates were identified and discarded. Titles and abstracts were screened and, for reports thus identified as potentially relevant, full texts were checked for eligibility. Studies were discarded where the full text was unavailable. Searching and determining the eligibility of target responses were carried out by three investigators independently.

### Analysis and data synthesis

The methods described here fulfilled the Preferred Reporting Items for Systematic Reviews and Meta-Analyses (PRISMA) guidelines (Liberati et al., 2009), as a meta-analysis was deemed inappropriate due to the heterogeneity of the examined study designs. To assess the risk of bias, the reviewers worked independently and determined the adequacy of the methodology with adequate reliability. Within the sample selected for review, studies were categorized by summarizing and comparing significant information and specifying the measures of the assessed variables for each study (see Table 1 for a detailed description of the reviewed studies).

**Table 1:** Distribution of the 17 relevant selected studies, including the reference, the population target, the aims, the measures of BED, the measures of suicidality, and the main results of the investigation.

**References**	**Population target and geographic location**	**Aims**	**Measures of BED**	**Measures of suicidality**	**Main results**
Ackard et al., 2011	*N* = 4,746 Adolescents with and without EDs, and subclinical EDs. United States	To examine substance use, depression, self-esteem, and suicidality by asymptomatic, subthreshold, and full threshold EDs diagnoses among nonclinical adolescents	Survey questions assessing EDs	Two questions about SI and SA	BED+ reported greater SI (OR 2.6) and SA (OR 3.1)
Ackard et al., 2003	*N* = 4,746 Adolescents with and without overeating, subclinical BED, and BED. United States	To determine the associations between overeating and sociodemographic characteristics, weight status, dieting behaviors, body satisfaction, depressive mood, self-esteem, and suicide	QEWP-R	Two questions about SI and SA	Among BED+ subjects, past SA was present in 28.6% of girls and 27.8% of boys, and SI levels were higher in girls (30.2%) than in boys (11.1%)
Annagur et al., 2015	*N* = 300 Obese sample with and without BED, and non-obese sample. Turkey	To evaluate depression and impulsivity in obese subjects with BED compared to non-obese healthy controls	SCID-I/P EAT	BDI	Lifetime SA was more prevalent in the obese group. BED associated with lifetime SA was present in 55.6% of obese patients, whereas SA in absence of BED diagnosis was present in 33.3% of obese patients
Carano et al., 2012	*N* = 80 Patients with BED. Italy	To evaluate the relationships between alexithymia and suicide ideation in a sample of outpatients with BED	SCID-I/P BEDCI BES	SSI	27.5% of BED+ reported SI. 61.3% of BED+ with alexithymia reported SI, whereas only 6.1% of BED+ without alexithymia reported SI 25.8% of BED+ with alexithymia reported SA, whereas only 4.1% of BED+ without alexithymia reported SA
Chen et al., 2008	*N* = 8 Female patients with BED or BN and BPD. United States	To provide summary data about the impact of standard DBT with minimal adaptation in patients with comorbid BED or BN and BPD	EDE	LPC SASII	No SA episodes in the course of treatment and during the 6-month follow-up period
Fichter and Quadflieg, 2016	*N* = 5,839 Patients with EDs. Germany	To report on long-term mortality, including the causes and predictors of early death in EDs	SIAB-S EDI	National register data on suicide completion	Among 65 dead patients with EDs, suicide was the cause of death in 4 patients with AN, 5 patients with BN, and 1 patient with BED
Forrest et al., 2017	*N* = 13,103 Adolescent and adult patients with EDs. United States	To assess the association between lifetime EDs and suicide ideation, planning, and attempting; namely, to investigate suicide risk in BED, and determine whether BED precedes suicidality, or vice versa	CIDI	Three items of CIDI about SI, SA, and suicide planning	BED was associated with elevated odds of SI, (adolescents: OR 3.81; adults aged 18–29: OR 4.05), SA (adolescents: OR 5.01; adults ORs 4.64–4.96), and suicide planning (adolescents: ORs 3.46–5.92). Furthermore, most adolescents experienced suicidality onset following BED onset, whereas most adults experienced suicidality onset prior to BED onset
Grucza et al., 2007	*N* = 910 Sample with and without BED. United States	To evaluate the prevalence and correlates of BED in a community sample	PHQ	One question about past SA	5.9% of obese BED+ reported past SA (OR 1.6); only 1.6% of obese BED-reported past SA (OR 3.7)
Izydorczyk and Mazur, 2012	*N* = 60 Female sample with and without BED. Poland	To measure the level of aggression and self-aggression among patients with BED and participants without eating or mental disorders	Survey questions assessing BED according to the ICD-10 criteria	IPSA-II	Significantly higher self-destructive tendencies (e.g., SI and SA) in BED+ than controls
McElroy et al., 2016	*N* = 1,092 Patients with BD. United States	To determine prevalence rates and clinical correlates of current DSM-5 EDs in patients with BD	EDDS	BiB-CQ	34% of patients with BD in comorbidity with BED reported past SA
McElroy et al., 2011	*N* = 875 Patients with BD. United States	To assess the prevalence and clinical correlates of EDs in patients with BD and the relationship of these disorders with demographic and illness variables	SCID-I/P	Survey questions about SA	The rate of suicide completion or SA among bipolar patients with BED was 11%
Pisetsky et al., 2013	*N* = 13,035 Female twins sample with and without EDs. United States/Sweden	To evaluate whether the prevalence of lifetime SA or completions was higher in women with a lifetime history of an ED than in women with no ED and to assess whether personality characteristics were associated with SA in women with EDs	Survey questions assessing BED according to the DSM-IV criteria	ICD-8, ICD-9, ICD-10 criteria	The rate of SA varied between 7.81 and 13.64% among BED+
Portzky et al., 2014	*N* = 1,436 Patients with EDs. Belgium	To examine associations between attempted suicide and trait and state-dependent characteristics in a large clinical population of ED patients	Interview assessing BED according to the DSM-IV criteria EDI-2	Three questions about SI and SA	51.7% of BED+ reported lifetime SI (OR 1.91); 10% of BED+ reported lifetime SA (OR 1.49)
Runfola et al., 2014	*N* = 2,269 Patients with EDs. United States	To examine the relation between self-image and SA or completions in women with EDs	SCID-I M.I.N.I. Kid SEDI EDE-Q	ICD-9, ICD-10 criteria for SA and suicide completion	6.7% BED+ reported past SA; these subjects showed worse self-image than BED+ without past SA
Suokas et al., 2014	*N* = 12,138 Patients with and without EDs. Finland	To explore the rates of hospital-treated SA among ED patients, and also to study predictors of SA and completed suicide	Survey questions assessing BED according to the DSM-IV criteria	ICD-9, ICD-10 criteria for SA	2.3% BED+ reported SA: the RR 2.66 was not significantly higher than controls
Swanson et al., 2011	*N* = 10,123 Adolescents with and without EDs, and subclinical EDs. United States	To describe the role impairment, suicidal behavior, and service use associated with EDs in adolescents	CIDI	Survey questions about SI, SA, and suicide planning	34.4% BED+ reported SI, 5.1% reported suicide planning, and 15.1% reported SA. Among adolescents with subclinical BED, 18.3% reported SI, 5.1% reported suicide planning, and 5.3% reported SA
Welch et al., 2016	*N* = 850 Patients with BED. Sweden	To explore clinical characteristics at diagnosis, diagnostic flux, psychiatric comorbidity, and SA in patients with BED	Interview assessing BED according to the DSM-IV criteria	ICD-9, ICD-10 criteria for SA and suicide completion	Rates of SA and suicide completion in BED+ were significantly high (5.8%). BED was associated with elevated risk for SA (OR 1.8)

*AN, anorexia nervosa; BED+, Patients with BED; BED, Patients without BED; BEDCI, Binge Eating Disorder Clinical Interview; BD, bipolar disorder; BES, Binge Eating Scale; BDI, Beck Depression Inventory; BiB-CQ, Bipolar Biobank Clinical Questionnaire; BN, bulimia nervosa; BPD, borderline personality disorder; CIDI, Composite International Diagnostic Interview - Version 3; DBT, dialectical behavioral therapy; DSM, Diagnostic and Statistical Manual of Mental Disorders; EAT, Eating Attitudes Test; ED, eating disorder; EDE, Eating Disorders Examination; EDE-Q, Eating Disorder Examination – Questionnaire; EDI, Eating Disorder Inventory; ICD, International Classification of Diseases; IPSA-II, Psychological Inventory of Aggression Syndrome; LPC, Lifetime-Parasuicide Count; M.I.N.I., Kid Mini International Neuropsychiatric Interview; PHQ, Patient Health Questionnaire; QEWP-R, Questionnaire on Eating and Weight Patterns-Revised; SA, suicide attempt; SASII, Suicide Attempt Self-Injury Interview; SCID-I/P, Structured Clinical Interview for DSM-IV Axis I Disorders – Patients Edition; SEDI, Structured Eating Disorder Interview; SI, suicide ideation; SIAB-S, Structured Inventory for Anorexic and Bulimic Eating Disorders Self-Rating Form; SSI, Scale of Suicide Ideation*.

## Results

**Figure 1:**
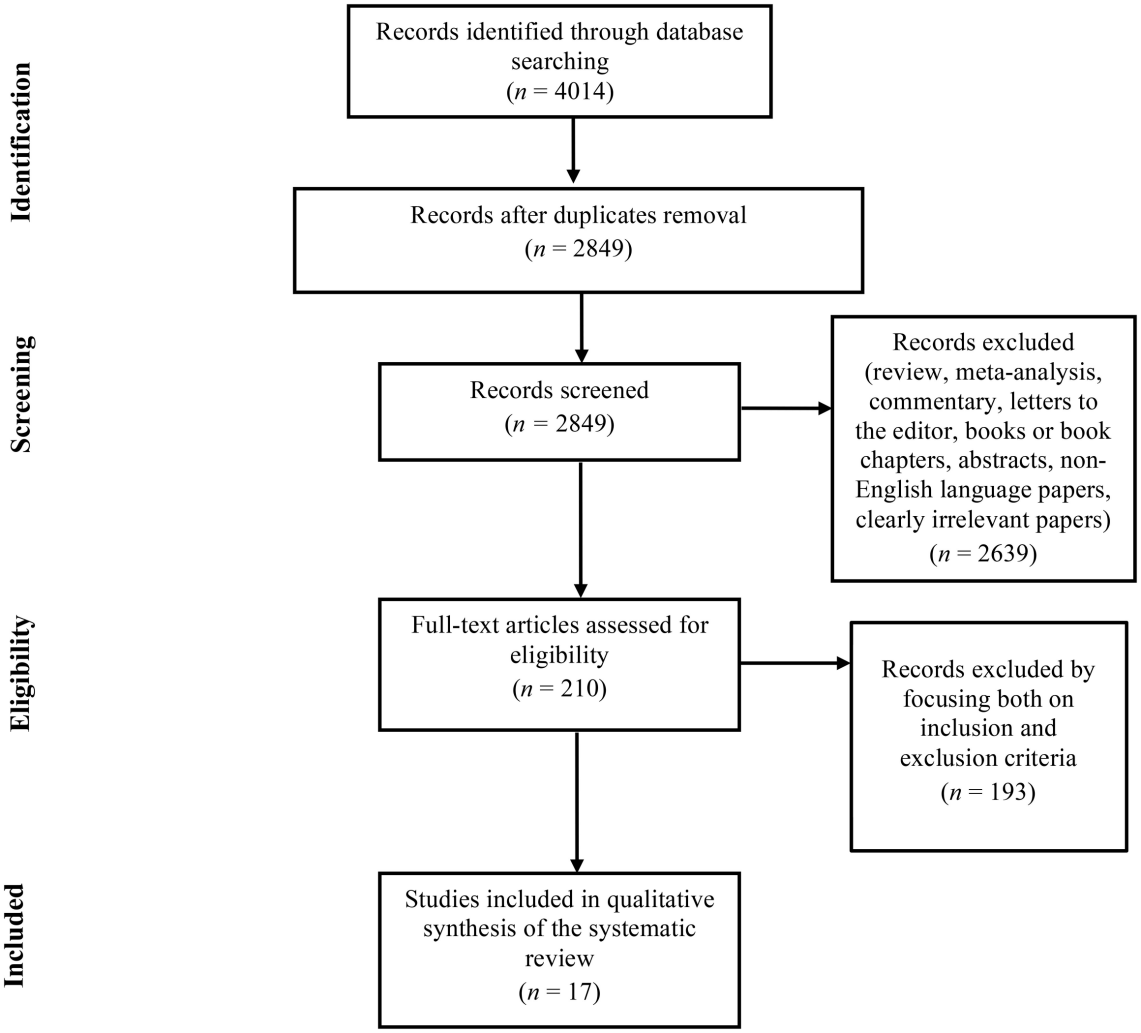
PRISMA Flowchart of the systematic search.

The search of electronic databases initially provided *n* = 4,014 citations, as reported in the PRISMA flowchart (Figure 1). After removing the duplicates, *n* = 2,849 records remained. Of these, *n* = 2,639 citations were eliminated as they were reviews, meta-analyses, commentaries, letters to the editor, books or book chapters, abstracts, non-English language papers, or because they did not meet the inclusion criteria. Of the 210 full text articles assessed for eligibility, *n* = 193 studies were excluded because they focused neither on inclusion nor exclusion criteria. Ultimately, *n* = 17 studies were selected for inclusion in the systematic review.

The reviewed studies were published between 2003 and 2016. These 17 papers reported the results of 12 cross-sectional analyses and 5 longitudinal analyses. In this section, the studies are grouped and described based on the characteristics of patients with BED at suicide risk.

### Epidemiological data on suicidality in bed

Among the studies investigating the associations between suicidality and BED, seven papers (Ackard et al., 2003, 2011; Swanson et al., 2011; Carano et al., 2012; Izydorczyk and Mazur, 2012; Portzky et al., 2014; Forrest et al., 2017) reported data on SI, whereas 16 papers reported data on SA and suicide completion in individuals with BED (Ackard et al., 2003, 2011; Grucza et al., 2007; Chen et al., 2008; McElroy et al., 2011, 2016; Swanson et al., 2011; Carano et al., 2012; Izydorczyk and Mazur, 2012; Pisetsky et al., 2013; Portzky et al., 2014; Runfola et al., 2014; Suokas et al., 2014; Annagur et al., 2015; Forrest et al., 2017; Welch et al., 2016). By examining the results of these studies, it was observed that the prevalence of SI varied between 26.3% (Ackard et al., 2003) and 51.7% (Portzky et al., 2014), and that the rates of SA and suicide completion varied between 2.3% (Suokas et al., 2014) and 34% (McElroy et al., 2016). Only a register-based study (Fichter and Quadflieg, 2016) provided specific data on suicide completion, reporting that suicide was the cause of death in 10 (1 of which suffering from BED) of 65 patients with an ED diagnosis.

### Clinical psychological characteristics of patients with bed at suicide risk

Studies of patients with BED have shown significant associations between suicidality and specific clinical psychological characteristics.

Carano et al. (2012) conducted a cross-sectional study to evaluate the relationship between alexithymia, SI, and SA in 80 adult outpatients with BED. In this sample, the prevalences of SI, SA, and alexithymia were 27.5, 12.5, and 38.8%, respectively. Patients with alexithymic traits reported more significant SI, a higher prevalence of current SI (61.3 vs. 6.1%), and more past suicide attempts (25.8 vs. 4.1%) compared with those without alexithymic traits. Furthermore, this study found that individuals with BED reported increased SI, especially in the presence of difficulty identifying feelings, difficulty describing feelings, and depressive symptoms, even if these symptoms are subclinical.

Izydorczyk and Mazur (2012) investigated the specific configuration of aggressive and self-aggressive tendencies (e.g., SA and suicidal thoughts) among female patients with BED, compared with healthy subjects. In this study, the clinical subjects showed significantly higher levels of aggressive and self-aggressive behaviors, such as suicidal thoughts and attempts.

Pisetsky et al. (2013), after furthering comprehension of suicide risk in women with lifetime EDs, confirmed an elevated risk of suicide in individuals with BED. In these patients, the prevalence of SA ranged between 7.81 and 13.64%. According to the study, patients with BED suffer from more serious neuroticism (as assessed with the Eysenck Personality Inventory) and show higher doubts about actions (as assessed with the Frost Multidimensional Perfectionism Scale). Moreover, in BED patients with a history of SA, lower levels of extraversion and self-directedness were observed in comparison with those without a history of SA, but these results are not statistically significant.

In a prospective study assessing self-image in female twins with EDs, 6.7% of patients reported BED and past SA. Moreover, this subgroup showed lower levels of self-affirmation, self-love, and self-protection, and higher levels of self-blame, self-hate, and self-neglect, than patients with BED without past SA (Runfola et al., 2014).

### Suicidality in bed among adolescents

Four of the studies examining the association between BED and suicidality involved adolescent populations.

In two studies, Ackard et al. (2003, 2011) recruited a school-based sample of 4,746 boys and girls in public middle and high schools, with the aim of examining associations between binge eating and sociodemographic characteristics, weight status, dieting behaviors, body satisfaction, depressive mood, self-esteem, and suicide. Both studies showed that adolescents meeting the criteria for BED scored significantly lower on measures of body satisfaction and self-esteem but higher on measures of BMI and depressive mood than those who reported subclinical BED or no binge eating. Moreover, the results suggested that SI in girls increases with the severity of binge eating symptoms (Ackard et al., 2003, 2011).

In the first study, Ackard et al. (2003) observed that 28.6% of girls and 27.8% of boys who met the criteria for BED reported past SA. Among adolescents with BED, they also found a higher level of SI in girls (30.2%), compared with boys (11.1%). In the second study, Ackard et al. (2011) found lower levels of self-esteem and higher prevalence of substance use, depressive mood, SI (OR 2.6), and SA (OR 3.1) in both the subthreshold and the threshold BED groups, in comparison with the asymptomatic groups (OR 1.0; OR 1.0).

Swanson et al. (2011) investigated SA and SI in a nationally representative sample of 10,123 adolescents aged from 13 to 18 years. They found that 1.6% (*n* = 164) and 2.5% (*n* = 253) of the sample suffered from BED or subthreshold binge eating, respectively. A total of 34.4% of the adolescents who met the criteria for BED reported SI, 15.1% had attempted suicide, and 5.1% had planned suicide. Moreover, it is important to highlight that in a subsequent and more recent paper (Forrest et al., 2017), this sample of adolescents was compared with 2,980 adults. This more recent study found that BED was associated with significantly elevated odds of SI among adolescents aged 13–18 (OR 3.81) and adults aged 18–29 (OR 4.05); BED was also associated with significantly elevated odds of suicide planning among adults (ORs 3.46–5.92) and of SA among adolescents (OR 5.01) and adults aged 18–29 (OR 4.64) and 45–59 (OR 4.96). Furthermore, adolescents affected by BED and suicidality experienced the onset of SI, planning, and/or attempting as subsequent to BED onset, whereas adults affected by BED and suicidality experienced the onset of SI, planning, and/or attempting prior to BED onset.

### Psychiatric risk factors for suicidality

Several studies have shown significant associations between BED, psychiatric disorders, and suicidality.

Grucza et al. (2007), who investigated the prevalence and correlates of BED in a community sample of 910 subjects, found that individuals with BED had nearly 4-fold higher odds of a history of one or more SAs (OR 3.7), sixfold higher odds of meeting major depression criteria (OR 5.4), and nearly fivefold higher odds of meeting the generalized anxiety disorder criteria (OR 5.3). Individuals with obesity who screened negative for BED exhibited no significant association with these outcomes.

Welch et al. (Izydorczyk and Mazur, 2012) investigated longitudinal data from the Swedish national ED registers and identified 850 individuals diagnosed with BED. The authors applied conditional logistic regression models to explore the association of BED and each comorbid psychiatric disorder with SA. Considerable diagnostic flux occurred across BED and other ED diagnoses. The strongest associations were found between BED and other EDs (OR 85.8), followed by those between BED and major depressive disorder (MDD) (OR 7.6), bipolar disorder (BD) (OR 7.5), anxiety disorders (OR 5.2), post-traumatic stress disorder (OR 4.3), and elevated risk of SA (OR 1.8). Depression and SA rates were elevated in individuals affected by BED with and without comorbid obesity.

In a study that compared 149 obese participants with 151 non-obese healthy controls, Annagur et al. (2015) found that a history of admission to psychiatric clinics and of depressive disorder were more prevalent among the obese group independently of the presence of BED. Lifetime SA was more prevalent among the obese group with BED (55.6%), whereas SA was present in 33.3% of obese group patients without BED. However, this is not a substantial difference, as the obese subjects with lifetime SA were only a few (*n* = 9).

Focusing specifically on mood disorders, McElroy et al. (2011, 2016) identified a high prevalence of BED in a sample of patients with BD in two studies conducted in 2011 and 2016, respectively. In the first study (McElroy et al., 2011), involving 875 patients with BD, BED was found to be the most common ED. The rate of suicide or serious SA among bipolar patients with BED was 11%.

Likewise, in the second study (McElroy et al., 2016) conducted on 1,092 bipolar spectrum patients, the authors found that 34% of BED patients had a history of SA. In this study, patients with BED were more likely to be women (72%), showed higher suicidality levels and greater anxiety disorder comorbidity, and had a higher mean BMI and a higher rate of obesity.

Finally, only a treatment developmental study provided data on the association between suicidality, BED, and personality disorders (Chen et al., 2008). The authors focused on the impact of standard dialectical behavior therapy (with minimal adaptation) on five patients with comorbidity of BED and borderline personality disorder. Before treatment, all the patients had a lifetime history of non-suicidal self-injury and/or suicidal behavior, including SA. At the post-treatment assessment, it was noticed that no SA episodes occurred in the course of treatment, and only one patient had attempted suicide during the 6-month follow-up period.

## Discussion

The present study aimed to systematically investigate published original research reports, evaluating the emerging clinical links between BED and suicidal factors.

The association between BED and suicide risk is significant, although there is a general paucity of studies investigating the effect size of BED on suicide risk, probably because BED has only recently been considered a nosographic category distinct from other EDs. In fact, only a few studies have analyzed data pertaining to individuals affected by BED separately from those pertaining to patients suffering other EDs.

As shown in the analysis of the collected articles, there is extreme variability in the methodologies used for investigating SI and SA. For example, in some of the articles analyzed, SI and SA were evaluated through only one question to subjects, leading to possible underestimation or overestimation of suicidality in the BED patient population (Ackard et al., 2003, 2011; Grucza et al., 2007; Portzky et al., 2014; Forrest et al., 2017). Furthermore, the data on suicide completion and on SA are not always reported separately, thus showing only the approximate incidence of mortality from suicide among patients in this clinical group (Pisetsky et al., 2013; Runfola et al., 2014; Welch et al., 2016).

BED has usually been associated with suicide risk when occurring with another psychiatric disorder, particularly MDD and BD (McElroy et al., 2011, 2016; Welch et al., 2016).

In studies analyzing both BED and other EDs, a similar risk level for SI, planning, and attempting is reported in individuals with BED as in those with anorexia and bulimia nervosa (Portzky et al., 2014; Suokas et al., 2014; Fichter and Quadflieg, 2016). These results suggest that the presence of BED, as well as other EDs, itself gives rise to suicide risk, as ED psychopathologies that implicate a self-injurious behavioral pattern. It is possible that increased SI levels may show a state-dependent phenomenon, possibly linked to greater BED severity (Ackard et al., 2003, 2011; Swanson et al., 2011). When considering BED as the tendency to engage in self-destructive behavior, Izydorczyk and Mazur (2012) demonstrated that patients with BED show more aggressive and self-aggressive behavior, including SA, than the general population. EDs and suicidality present the same risk factors, including body dissatisfaction (Kim and Kim, 2009; Nolen-Hoeksema and Watkins, 2011), interoceptive deficits (Forrest et al., 2015), and emotion dysregulation (Stice, 2002). Alternatively, environmental risk factors, such as physical abuse, could be considered important risk factors for both BED (Copeland et al., 2015) and suicidal behavior (Johnson et al., 2002; Bruffaerts et al., 2010). A factor such as impulsivity could represent a genetically influenced intermediate phenotype behind both BED (Schag et al., 2013; Hege et al., 2015) and some manifestations of suicidal behavior (Anestis et al., 2014; Rimkeviciene et al., 2015). To this extent, Wade et al. (2015) hypothesize a possible common genetic basis for suicidality and EDs, highlighting the need for deepened investigation of whether these common factors may be linked to emotional dysregulation or to other temperament factors.

Only a few studies have investigated the personality factors in BED patients with suicidal behaviors. Although the data are contrasting, personality factors seem to identify the subgroups of patients with BED at higher risk of suicide (Carano et al., 2012; Pisetsky et al., 2013; Runfola et al., 2014; Annagur et al., 2015). Recognizing this may improve psychotherapy treatment for suicide risk, as shown by Chen et al. (2008) in a study that, though investigating a small sample, underlines how treating patients with BED and borderline personality disorder reduces suicide risk.

Overweight and obese individuals are also at high risk of suicide, but data on the role of BED in predicting suicide risk in this clinical population are inconsistent, as the samples examined by the studies reviewed herein were too small to be statistically significant (Jenkins et al., 2002; Annagur et al., 2015; Fichter and Quadflieg, 2016).

Adolescents with BED may represent another subgroup at suicide risk requiring special attention. In studies focusing on this population, adolescents with BED are shown to exhibit a higher level of suicide risk compared with the general adolescent population (Ackard et al., 2003, 2011; Swanson et al., 2011). These results underline the need to deepen knowledge of the manifestations of BED symptomatology from a lifetime perspective. Only Forrest et al. (2017) have conducted investigations into suicidality onset in BED. According to their study, while suicidality manifested after BED onset in the adolescent population, the reverse was found in the adult population. This may be explained by the fact that if binge eating starts during adolescence, the possibility of more severe psychopathologies and later negative outcomes (e.g., suicide) increases (Forrest et al., 2017).

In conclusion, BED is associated with elevated risk of suicide. This association may be accounted for by comorbid psychopathology or by the commonalities present in both BED and other psychiatric conditions. The findings demonstrate that BED is a severe ED and needs comprehensive treatment to prevent suicidal behavior. They also underline the need for intensive treatment and monitoring.

The present review supports the need to establish the psychological processes that may induce SI and suicidal behavior in BED. From a clinical perspective, it seems essential to identify those subgroups at higher suicide risk through their psychological characteristics, psychopathologies, and BED onset. To most effectively prevent and treat suicidality among patients affected by BED, it should be examined how BED, comorbid psychopathology, and suicidality are interrelated and affect one another over time.

## Author contributions

CC wrote the paper and provided substantial contributions to the conception and design of the review paper. RL and MS wrote the manuscript and conducted the computer search by selecting clinical relevant research articles. MI wrote the paper by revising it critically for important intellectual content. MG gave clinical suggestions for the paper. MF gave final approval of the version to be submitted. Finally, all the authors have approved the final version of the manuscript and were accountable for the content of the work.

### Conflict of interest statement

The authors declare that the research was conducted in the absence of any commercial or financial relationships that could be construed as a potential conflict of interest. The reviewer, RB, and handling Editor declared their shared affiliation.
